# Anti-inflammatory Therapy for Coronary Atherosclerotic Heart Disease: Unanswered Questions Behind Existing Successes

**DOI:** 10.3389/fcvm.2020.631398

**Published:** 2021-02-01

**Authors:** Jun Ma, Xiaoping Chen

**Affiliations:** ^1^Department of Cardiology, West China Hospital, Sichuan University, Chengdu, China; ^2^Department of Cardiology, The General Hospital of Western Theater Command, Chengdu, China

**Keywords:** atherosclerotic heart disease, interleukin 1β (IL-1β), interleukin 6 (IL-6), colchicine, anti-inflammatory therapy

## Abstract

Coronary atherosclerotic heart disease is a serious threat to human health. The results of the Canakinumab Anti-Inflammatory Thrombosis Outcome Study published in 2017 put an end to the perennial debate about the anti-inflammatory treatment of coronary atherosclerotic heart disease. In addition to interleukin 1β monoclonal antibody, interleukin 6 receptor antagonists and colchicine have also shown exciting results in clinical trials within the last 3 years. However, behind these successes, questions remain that need to be addressed. In this review, we summarize the successes and existing doubts of interleukin 1β antibodies, interleukin 6 receptor antagonists, and colchicine in the anti-inflammatory treatment of coronary atherosclerotic heart disease.

The occurrence and development of coronary atherosclerotic heart disease are thought to be associated with a variety of risk factors, including hypertension, hyperlipidemia, diabetes mellitus, smoking, etc. (1, 2). Prevention and control of coronary heart disease through strict control of risk factors can be significantly effective (3). However, a previous study showed that even in low-risk populations without appealing risk factors, a significant proportion of the population still suffered from coronary atherosclerotic heart disease (4). On the other hand, standard treatment after a percutaneous coronary intervention (PCI) failed to inhibit the progression of non-offending coronary plaque (5). Therefore, the treatment of coronary artery disease still needs further refinement.

Inflammation is an important contributor to the development and progression of atherosclerotic plaque (6–8); diseases characterized by chronic inflammation (for example, rheumatism and gout) are disproportionately burdened by coronary atherosclerotic heart disease (9–12). Analyses of randomized human trials showed that patients with coronary artery disease could further benefit from lowering low-density cholesterol (LDL-C) if they also reduce the risk of inflammation (13–16), and statins were highly effective in patients with elevated high-sensitivity C-reactive protein (hsCRP) and low levels of LDL-C (17, 18). As evidence from basic and clinical studies accumulates, many researchers believe that anti-inflammatory therapy could be a new option to break the bottleneck in the treatment of atherosclerotic heart disease. Thus, researchers have conducted a series of clinical trials on inflammation and coronary artery disease. The results showed that anti-inflammatory therapy might become an important tool for optimizing existing coronary drug therapy. However, behind the success, there are still questions. This article summarized the successes and unanswered questions of current anti-inflammatory treatments for coronary atherosclerotic heart disease and provided a discussion.

## Path to Success in Clinical Studies

A series of clinical trials have been conducted in recent years for the anti-inflammatory treatment of coronary atherosclerotic hearts, including but not limited to Anti-inflammatory Therapy with Canakinumab for Atherosclerotic Disease (CANTOS) (19), Low-Dose Methotrexate for the Prevention of Atherosclerotic Events (20), Low-Dose Colchicine for Secondary Prevention of Cardiovascular Disease (LoDoCo) (21), Colchicine in Patients with Chronic Coronary Disease (LoDoCo2) (22), etc. These clinical trials can be divided into two categories (Table 1): therapy with a clear target (most are inflammation-related cytokines), such as CANTOS, and broad-spectrum anti-inflammatory approach, such as LoDoCo series trials and Low-Dose Methotrexate for the Prevention of Atherosclerotic Events. From the results so far, the drugs targeting interleukin (IL)-6 receptor/IL-1β have achieved positive results, whereas the low dose of colchicine is the most promising for the broad-spectrum anti-inflammatory approach.

**Table 1:** Major completed clinical trials involving anti-inflammatory agents in atherosclerotic heart disease.

**Trial name**	**Study design**	**Study population**	**Patient number**	**Follow-up (years)**	**Intervention**	**Target**	**Primary outcomes**	**Result**
**Therapy with a clear target**								
Effect of a single dose of the interleukin-6 receptor antagonist tocilizumab on inflammation and troponin T release in patients with non-ST-elevation myocardial infarction (23)	Phase II, two-center, double-blind, placebo-controlled trial	Patients with NSTEMI	117	0.5	Tocilizumab 280 mg prior to coronary angiography vs. Placebo	Il-6ra	Auc for hscrp during hospitalization	4.2 mg/L/h (placebo) vs. 2.0 mg/L/h (tocilizumab); p < 0.001
ASSAIL-MI (24)	Phase II randomized, double blind, placebo-controlled trial	Patients with first-time STEMI presenting within 6 h of the onset of chest pain	199	0.5	Tocilizumab 20 mg/ml once vs. Placebo	Il-6ra	Myocardial salvage index	Completed, results not published
MRC-ILA Heart (25)	Phase II, double-blinded, randomized, placebo-controlled	Patients with NSTE-ACS, presenting <48 h from onset of chest pain	182	1.0	100 mg of anakinra once daily vs. Placebo	Il-1ra	Auc for c-reactive protein over the first 7 days	IL-1Ra group, 21.98 mg day/L (95%CI 16.31–29.64) vs. placebo group, 43.5 mg day/L (31.15–60.75); *p* = 0.0028
CANTOS (19)	Phase III multicenter, randomized, double-blind, placebo-controlled	Patients with MI and elevated hsCRP	10,061	2.1	Canakinumab (50, 150, or 300 mg) every 3 months vs. Placebo	Il-1β	Non-fatal mi, non-fatal stroke and cardiovascular death	HR 0.85;95% CI 0.75–0.98; *p* = 0.021 in 150 mg- treated group
LATITUDE-TIMI 60 (26)	Phase III multicenter, randomized, double-blind, placebo-controlled	Patients had been hospitalized with a presumed spontaneous (type 1) MI	3,503	0.4	Twice-daily losmapimod 7.5 mg vs. Placebo	Mapk	Composite of cardiovascular death, mi, or severe recurrent ischemia requiring urgent coronary revascularization	HR 1.16; 95% CI, 0.91–1.47; *p* = 0.24
SOLID-TIMI 52 (27)	Phase III multicenter, randomized, double-blind, placebo-controlled	Patients within 30 days of hospitalization with an ACS	13,026	2.5	Once-daily darapladib 160 mg vs. Placebo	Lp-pla_2_	Composite of coronary heart disease (chd) death, mi, or urgent coronary revascularization for myocardial ischemia	HR 1.00;95% CI, 0.91–1.09; *p* = 0.93
**Broad-spectrum anti-inflammatory approach**								
CIRT (20)	Phase III multicenter, randomized, double-blind, placebo- controlled trial	Patients with previous myocardial infarction or multivessel coronary disease who additionally had either type 2 diabetes or the metabolic syndrome	4,786	2.3	Methotrexate (target dose of 15–20 mg weekly) vs. Placebo	Multiple	Non-fatal mi, non-fatal stroke and cardiovascular death	HR 1.01; 95% CI 0.82–1.25; *p* = 0.91
LoDoCo (21)	Prospective, randomized, observer-blinded	Patients with stable coronary disease	532	3.0	Colchicine 0.5 mg/day vs. Placebo	Multiple	Composite incidence of acute coronary syndrome, out-of-hospital cardiac arrest, or non-cardioembolic ischemic stroke	HR 0.29; 95% CI: 0.15 to 0.56; *p* < 0.001
COLCOT (28)	Phase III multicenter, randomized, double-blind, placebo- controlled trial	Patients recruited within 30 days after a myocardial infarction	4,745	1.8	Colchicine 0.5 mg/day vs. Placebo	Multiple	Composite of death from cardiovascular causes, resuscitated cardiac arrest, myocardial infarction, stroke, or urgent hospitalization for angina leading to coronary revascularization	HR 0.77; 95% CI, 0.61 to 0.96; *p* = 0.02
LoDoCo 2 (22)	Phase III multicenter, randomized, double-blind, placebo- controlled trial	Patients with chronic coronary disease	5,522	2.3	Colchicine 0.5 mg/day vs. Placebo	Multiple	Composite of cardiovascular death, spontaneous (nonprocedural) myocardial infarction, ischemic stroke, or ischemia-driven coronary revascularization	HR 0.69; 95% CI, 0.57 to 0.83; *p* < 0.001
COPS (29)	Multicenter, randomized, double-blind, placebo-controlled trial	Patients with acs and had evidence of coronary artery disease on coronary angiography	795	1.0	Colchicine 0.5 mg twice daily for first month, then 0.5 mg daily for 11 months	Multiple	Composite of all-cause mortality, acs, ischemia-driven (unplanned) urgent revascularization and non-cardioembolic ischemic stroke	HR 0.65; 95% CI, 0.38 to 1.09; *p* = 0.10

*CI, confidence interval; HR, hazard ratio; IL, interleukin; MI, myocardial infarction; Ra, receptor antagonist; AUC, area under the curve; NSTEMI, non-ST-segment elevation myocardial infarction; STEMI, ST-segment elevation myocardial infarction; ACS, acute coronary syndrome; MAPK, p38 mitogen-activated protein kinase; Lp-PLA2, lipoprotein-associated phospholipase A2*.

Historically, the evidence for the involvement of inflammation in the formation of atherosclerosis has been accumulated for more than 30 years (30, 31). Although basic research suggests that activation of many inflammatory factors or inflammatory pathways promotes the development of atherosclerotic disease (30), the current clinical findings suggest that the key point of anti-inflammatory treatment of atherosclerotic disease is most likely present in the IL-1β/IL-6/C-reactive protein (CRP) pathway. Tracing the footsteps of several successful clinical trials to date, we can divide the history of research development in anti-inflammatory therapy for atherosclerotic heart disease into three stages: from 1990 to 1999, the work done by researchers was mainly observational research trials in which they discovered the important predictive role of CRP in the occurrence of adverse cardiovascular events in patients with coronary heart disease (32–35); then from 2000 to 2009, researchers began to look for further clues linking inflammation to coronary heart disease and tried to find suitable therapeutic targets. They found that CRP is better suited as a biomarker than a therapeutic target, and instead, the key CRP upstream protein, IL-6, is a potential therapeutic target (36–38); over the past decade, researchers have conducted a series of clinical drug trials for the anti-inflammatory treatment of coronary atherosclerotic heart disease and have achieved important results. Among them, the drugs with positive results are mainly IL-1β monoclonal antibodies, IL-6 receptor antagonists (IL-6Ra), and the traditional anti-inflammatory drug colchicine.

## What Unanswered Questions Lie Behind the Success?

### Interleukin-6 Receptor Antagonist in the Treatment of Coronary Atherosclerotic Heart Disease

IL-6 is the main upstream protein of CRP (39). Basic studies have shown that IL-6 is associated with plaque initiation and instability (40). CRP or hsCRP is an important marker for predicting the risk of cardiovascular events in coronary atherosclerotic heart disease. Although lowering CRP levels can improve outcomes in patients with coronary heart disease (41), genetic polymorphisms in CRP in the population are not significantly associated with disease risk of ischemic vascular disease (36), and researchers consider CRP itself is unlikely to provide an effective target for intervention (39). For these reasons, researchers moved to IL-6; they found that elevated levels of IL-6 are associated with increased risk of future myocardial infarction (MI) in healthy men (42) and treatment with tocilizumab (IL-6Ra) significantly attenuates inflammation and PCI-related troponin T release in patients with acute non-ST-elevation myocardial infarction (NSTEMI) (23). Mendelian randomization studies demonstrate that polymorphism in the IL-6 signaling pathway at rs2228145 and rs7529229 concordantly associate with both lifetime lower levels of hsCRP and lifetime lower risks of coronary heart disease (40, 43). Next, researchers designed a clinical trial to evaluate the effect of tocilizumab on myocardial salvage in patients with acute ST-elevation myocardial infarction (STEMI), and this clinical trial is called the ASSAIL-MI trial. The trial was a multicenter randomized controlled trial that ultimately included 199 subjects, randomly assigned 1:1 to a tocilizumab treatment group and a placebo group; subjects in both groups assess the efficacy of treatment at 3–7 days, 3 months, and 6 months of follow-up, respectively (24). The results published in European Society of Cardiology Congress 2020 showed that treatment of patients after first acute STEMI with tocilizumab significantly improved the myocardial salvage index. In a prespecified subgroup analysis, it was found that patients with ischemic episodes lasting longer than 3 h before PCI had a more significant effect with tocilizumab. Therefore, IL-6 is an important anti-inflammatory therapeutic target for MI patients.

### Efficacy and Safety of Long-Term Use of Interleukin-6 Receptor Antagonists Needs to Be Further Evaluated

The clinical evidence for IL-6Ra is insufficient compared with IL-1β and colchicine. There are no large randomized controlled studies of IL-6Ra with major cardiovascular events as endpoints. The long-term prognostic effect of IL-6 antibodies in coronary atherosclerotic disease is unclear. A potential limitation of long-term treatment with direct IL-6 inhibition is that this approach may upregulate apolipoprotein B, leading to an increase in LDL-C, and this effect was dose-dependent, potentially unrelated to inflammatory status, and thus a significant limiting factor in the development of IL-6 receptor blockade for atherosclerosis (44–46). In fact, the possible actions for IL-6 involved in coronary atherosclerotic heart disease included initiation and progression of atherogenesis (47), the stability of atherosclerotic plaques (48), thrombosis (49), myocardial ischemia/reperfusion injury, ventricular remodeling, and development of heart failure (50, 51). Researchers who designed the ASSAIL-MI trial also focused on the effect of tocilizumab in ischemia/reperfusion injury and myocardial remodeling after MI but did not address the atherogenesis and stability of atherosclerosis (24). Thus, it seems that from the current clinical studies that anti-IL-6 primarily acts on the myocardium rather than on vascular plaques.

In addition, although no significant adverse effects were observed with IL-6Ra at 6 months (23), the safety of long-term tocilizumab use was not fully assured. The CANTOS clinical trial showed that the incidence of fatal infections and sepsis was significantly higher in the canakinumab (a therapeutic monoclonal antibody targeting IL-1β) group, and the IL-6 levels in the canakinumab group were significantly lower than baseline (19). Considering the anti-inflammatory effects of IL-6 (52–55), the long-term safety of IL-6Ra still needs to be validated by clinical trials with long-term follow-up.

### Therapeutic Monoclonal Antibody Targeting Interleukin-1β in Treatment of Coronary Atherosclerotic Heart Disease

One of the important upstream pathways of IL-6 is the IL-1 signaling pathway. It has been proved that deficiency in or delivery of an antagonist of the receptor for IL-1 (IL-1R) in mice unequivocally results in reductions in atherosclerosis (56). In clinical trials, although IL-1Ra treatment reduced inflammatory markers in NSTEMI patients at 14 days after NSTEMI, major adverse cardiovascular events at 30 days and 3 months were similar in the IL-1Ra group as in the control group and occurred at a higher rate at 1 year (25). IL-1β is the primary circulating form of IL-1 and then has been much focused; basic studies have shown that IL-1β deficiency decreases the severity of atherosclerosis (57). CANTOS is the first large-scale clinical trial to prove the effectiveness of anti-inflammatory treatment for coronary atherosclerotic heart disease and the inflammation hypothesis of atherosclerotic disease. The trial enrolled 10,061 patients with previous MI and hsCRP level of 2 mg or more per liter and compared three doses of canakinumab (50, 150, and 300 mg, administered subcutaneously every 3 months) with placebo. The results show that canakinumab significantly reduced hsCRP level, as compared with placebo, without reducing the LDL-C level, and the 150-mg dose resulted in a significantly lower incidence of recurrent cardiovascular events than placebo after 2-year follow-up (19). After a median of 3.7 years of follow-up, canakinumab reduced the rates of total serious cardiovascular events, with rates per 100 person-years in the placebo, 50-, 150-, and 300-mg canakinumab groups of 10.4, 8.4, 8.3, and 8.2, respectively (58). The results suggest that all three doses of canakinumab can work.

### Clinical Use of Interleukin-1β Monoclonal Antibody Faces Difficulties

The IL-1β monoclonal antibody in the treatment of coronary atherosclerotic heart disease is also facing some problems. Despite the positive results with CANTOS, canakinumab was not approved for clinical use in patients so far. This may be due to the high price of canakinumab as well as the risk of serious infection (19). More recently, the CANTOS trial group reported that there remains a substantial residual inflammatory risk related to both IL-18 and IL-6 after IL-1β inhibition with canakinumab (59). Therapeutic monoclonal antibody targeting IL-1β confirms the inflammatory theory of atherosclerotic heart disease but is still far from clinical treatment.

Additionally, researchers who designed CANTOS believe that canakinumab reduces atherothrombosis by acting directly on IL-1β in the circulation (39), but basic research shows that IL-1β promotes multiple beneficial changes in late-stage murine atherosclerosis, including promoting outward remodeling and formation and maintenance of a smooth muscle cell/collagen-rich fibrous cap (60). Therefore, the role of IL-1β in atherosclerotic plaques remains to be discussed.

### Colchicine in Treatment of Coronary Atherosclerotic Heart Disease

From the results of current studies, colchicine is the most promising drug for clinical use in the treatment of coronary atherosclerotic heart disease. As a common anti-inflammatory drug (61, 62) for the treatment of acute gout and rheumatism, colchicine not only has the advantage of being inexpensive and safe (63–66) but also shows a protective effect against cardiovascular diseases in gout patients. A retrospective study reported in 2012 show that gout patients who took colchicine had a significantly lower prevalence of MI vs. those who did not take colchicine (1.2 compared with 2.6%, *P* = 0.03; 54% relative risk reduction) (67), and Solomon et al. (68) show that colchicine used in gout patients was associated with a 49% relative risk reduction in a composite primary outcome of MI, stroke, and transient ischemic attack compared with patients who did not use colchicine, as well as a 73% relative risk reduction in all-cause mortality. The potential for treating coronary atherosclerotic heart disease demonstrated by colchicine has attracted researchers to design a series of relevant clinical trials. Stefan et al. (21) designed a prospective, randomized, observer-blinded clinical trial and found that the addition of 0.5-mg colchicine daily significantly reduced adverse cardiovascular events in patients with stable coronary artery disease under regular secondary prevention therapies compared with no addition of colchicine (4.5 vs. 16.0%; hazard ratio: 0.29; 95% CI: 0.15 to 0.56; *p* < 0.001), and this trial is also called the LoDoCo study. Building upon the positive result, researchers conducted the LoDoCo2 study, a phase 3 multicenter, double-blind, randomized placebo-controlled clinical trial. The LoDoCo2 randomized 5,522 patients with stable atherosclerosis to receive either 0.5 mg/day of colchicine or a matching placebo in addition to proven secondary prevention therapies. After an average follow-up of 28.6 months, the result demonstrates that 0.5 mg of colchicine once daily resulted in a 31% lower relative risk of the primary endpoint (cardiovascular death, spontaneous MI, ischemic stroke, or ischemia-driven coronary revascularization) than placebo, with a hazard ratio of 0.69 (22). In addition, colchicine also has a protective effect in post-MI patients. The Efficacy and Safety of Low-Dose Colchicine after Myocardial Infarction [COLCOT clinical trial (28)] indicates that colchicine at a dose of 0.5 mg daily led to a significantly lower risk of ischemic cardiovascular events than placebo (primary endpoint occurred 5.5 vs. 7.1%; hazard ratio, 0.77; 95% CI, 0.61 to 0.96; *P* = 0.02) among patients with a recent MI. Several meta-analyses have shown that colchicine reduces the risk of future cardiovascular events, including stroke, in patients with coronary heart disease (69–72). It seems that colchicine may be the fastest drug available for clinical use in the treatment of coronary atherosclerotic heart disease.

### Anti-inflammatory Effects of Colchicine in Atherosclerotic Heart Disease Need Further Evaluation

The result from the COPS clinical trial, a multicenter, randomized, double-blind, placebo-controlled trial published on August 29, 2020, that showed the addition of colchicine to standard medical therapy did not significantly affect cardiovascular outcomes at 12 months in acute coronary syndrome (ACS) patients and was associated with a higher rate of mortality (29). Over the 12-month follow-up, there were 24 events in the colchicine group compared with 38 events in the placebo group experiencing the event (*p* = 0.09, log-rank). There was a higher rate of total death in the colchicine group (8 vs. 1, *p* = 0.017, log-rank). This result is surprising because ACS has higher levels of inflammation than chronic coronary syndrome (73, 74). Theoretically, by reducing the inflammatory burden, colchicine is more effective in patients with ACS.

**Figure 1:**
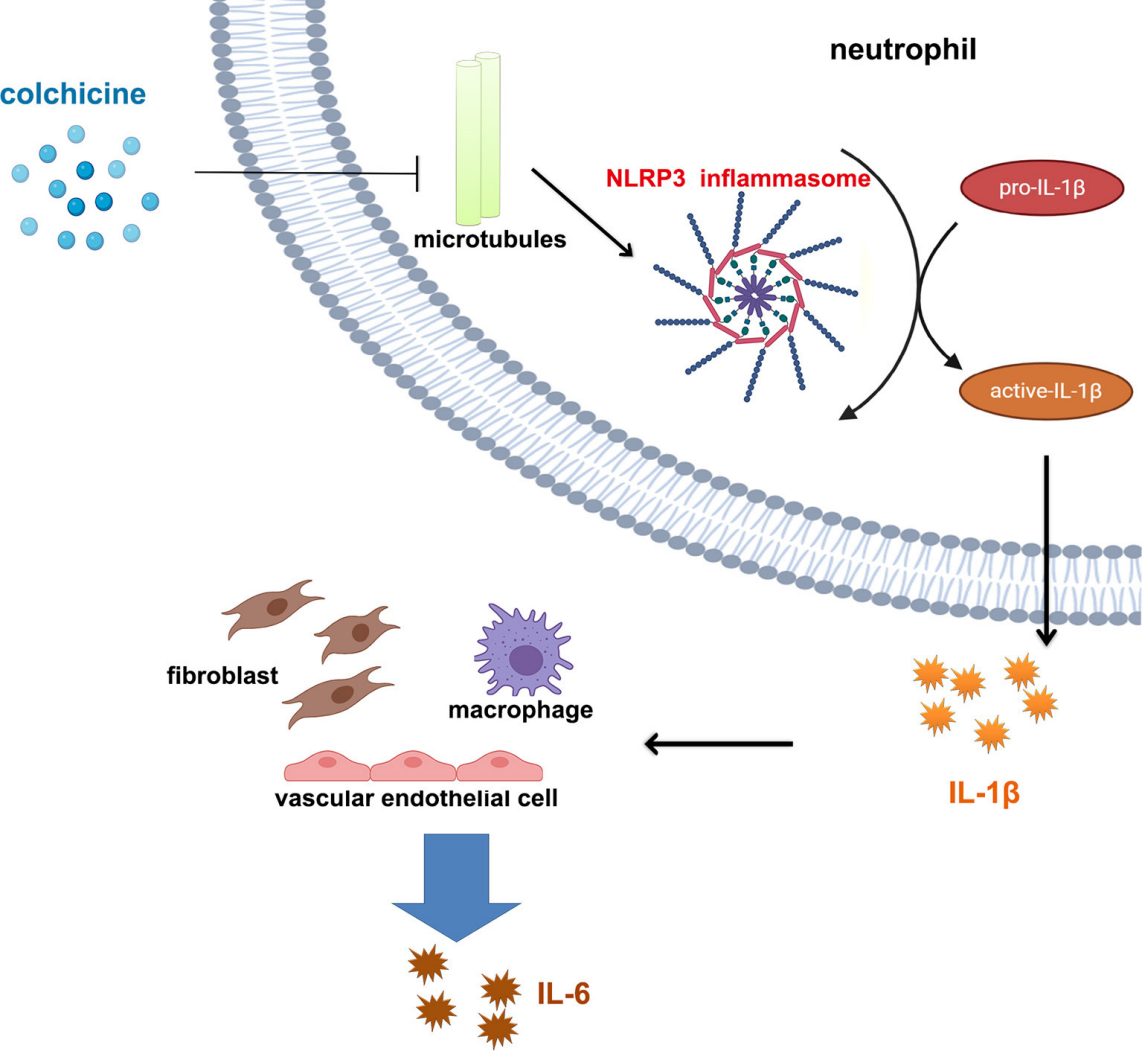
Colchicine affects interleukin 1β/interleukin 6 pathway through NLRP3 inflammasome. Colchicine prevents assembly of NLRP3 inflammasome in cells by inhibiting the function of microtubules, thus reducing the maturation and release of IL-1β and further reducing release of IL-6 from various types of cells.

In fact, the anti-inflammatory effects of colchicine in coronary atherosclerotic heart disease have not been effectively evaluated. In current studies, changes in levels of inflammation (e.g., CRP, IL-6, etc.) were not assessed in either LoDoCo, LoDoCo2, or COLCOT. Results from LoDoCo-MI (a pilot randomized placebo-controlled trial of colchicine after acute MI) shows that treatment with 0.5 mg/day of colchicine was not associated with a significantly increased likelihood of achieving a CRP level <2 mg/L or lower absolute levels of CRP 30 days after an acute MI (75). In terms of the mechanism, colchicine is a dose-dependent, broad-spectrum anti-inflammatory drug (64), and colchicine inhibits inflammation *via* NOD-like receptor family pyrin domain containing 3 (NLRP3) (NOD-, leucine-rich repeats-, and pyrin domain-containing protein 3); inflammasome is believed to act as a key mechanism in coronary arteriosclerotic heart disease (76–79). NLRP3 is an intracellular sensor, and the activation of NLRP3 inflammasome leads to caspase 1-dependent release of the pro-inflammatory cytokines IL-1β (80). Studies showed that colchicine inhibits NLRP3 inflammasome assembly by inhibiting cellular microtubules function (81–83). Thus, it is hypothesized that the use of colchicine can affect IL-1β and downstream levels of IL-6 and CRP *via* NLRP3 inflammasome (Figure 1), especially for patients after MI (77). Some researchers concluded that NLRP3 protein might not play a role in the acute development of MI due to low cardiac expression (84), which may explain the failure of colchicine to take positive results in patients with ACS. However, more evidence has proven that NLRP3 inflammasome-mediated inflammation does have an important role in ACSs (77, 85). On the other hand, studies have also shown that colchicine does not necessarily activate NLRP3 (86). Therefore, the anti-inflammatory effects of colchicine in atherosclerotic heart disease, especially in MI, need to be further evaluated.

Another point that still needs to be investigated is whether colchicine plays an anti-inflammatory role independent of blood lipid levels. A previous study (87) have demonstrated that continuous oral administration of colchicine (1 mg/day) for 1 year reduces the level of the oxidized low-density lipoprotein in patients with primary biliary cirrhosis, and a recent animal study (88) found a synergistic lipid-lowering effect of colchicine in combination with statin for a short period (2 weeks). The LoDoCo, LoDoCo2, or COLCOT studies all used secondary prevention of coronary heart disease (including statins), but none evaluated the effect of colchicine on lipids. Thus, the possibility that colchicine acts on coronary atherosclerotic heart disease by lowering blood lipids cannot be completely ruled out.

## Summary

In recent years, anti-inflammatory treatment of coronary atherosclerotic heart disease has achieved a stage victory. Inflammatory factor-targeted drugs such as canakinumab have confirmed the inflammatory theory of coronary heart disease, and classical anti-inflammatory drugs such as colchicine have demonstrated the possibility of rapid clinical use.

However, behind the success, there are still some questions that need to be addressed: canakinumab is expensive, has serious adverse effects, and remains a residual inflammatory risk; tocilizumab has an effect on lipid levels, its long-term effects and safety are unknown, and it is expensive; anti-inflammatory effects of colchicine in atherosclerotic heart disease needs further evaluation. Moreover, during ischemia, the level of inflammation in the myocardium is also increased, thereby damaging cardiomyocytes (89), and in the case of MI, the inflammatory response triggered by dead myocardial cells further increases the size of the infarct (90–92). If the effective effects of anti-inflammatory therapy are dominated by limiting the inflammatory response of ischemic or infarcted cardiomyocytes (e.g., IL-6Ra), the use of anti-inflammatory drugs in other atherosclerotic diseases (e.g., stroke, peripheral vascular disease, etc.) may be limited.

**Figure 2:**
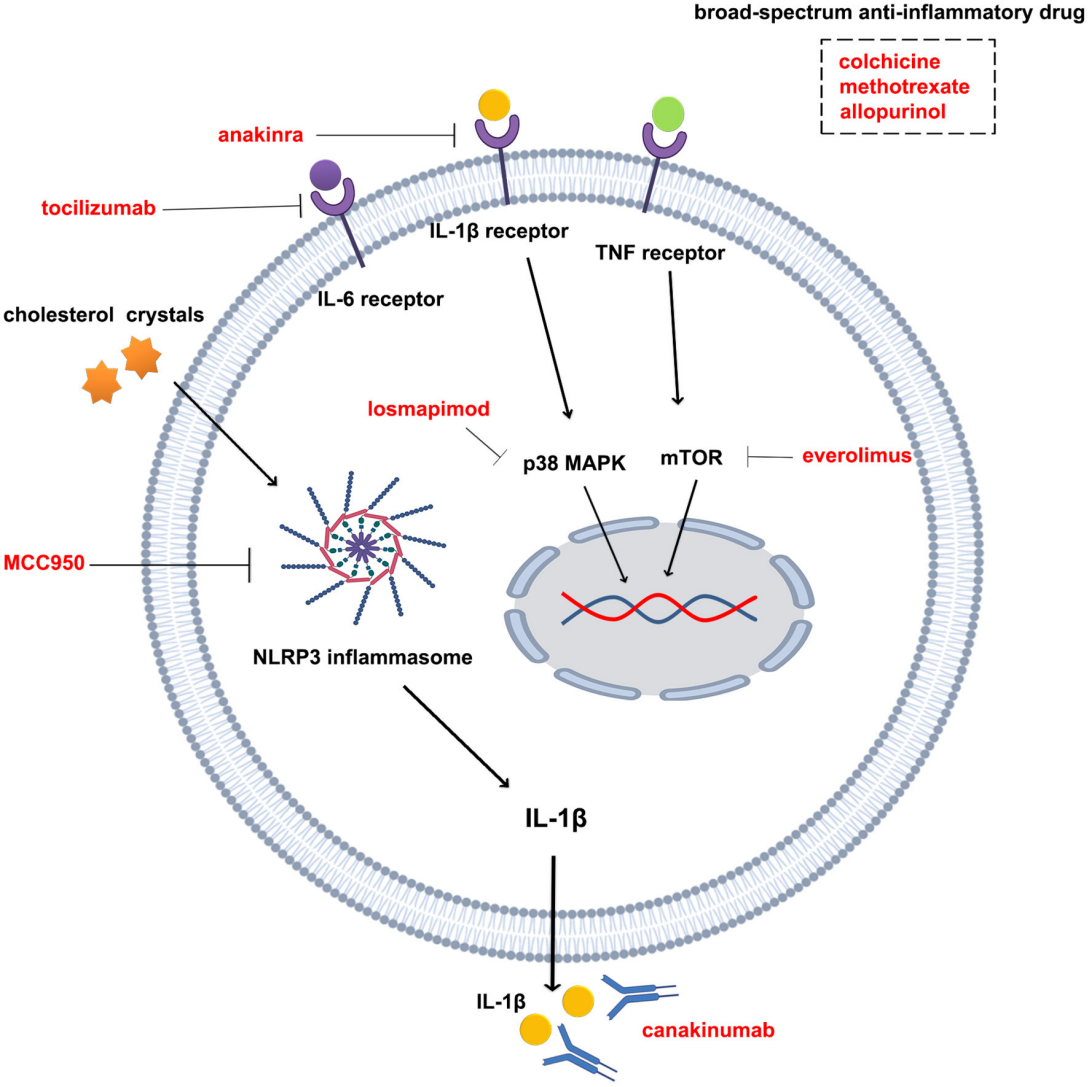
Drugs and targets for anti-inflammatory therapy in atherosclerotic diseases. Tocilizumab is an interleukin-6 receptor antagonist; anakinra is an interleukin 1β receptor antagonist; canakinumab is an interleukin 1β monoclonal antibody; losmapimod is a p38 MAPK inhibitor; everolimus is an mTOR inhibitor; MCC950 is an NLRP3 inflammasome inhibitor; colchicine, methotrexate, and allopurin are broad-spectrum anti-inflammatory drugs.

What we can look forward to is that many studies related to the anti-inflammatory treatment of atherosclerotic disease are ongoing (Table 2 and Figure 2), including the evaluation of the protective effect of colchicine against cerebral ischemia, another clinical trials of colchicine in ACS and other anti-inflammatory drugs (e.g., Allopurinol, EVERolimus). These subsequent studies will provide strong evidence for determining the feasibility of anti-inflammatory therapy in coronary heart disease and even in atherosclerotic disease. In addition, NLRP3 inflammasomes are closely associated with colchicine, IL-1β, and IL-6 and are thought to be potentially important targets for anti-inflammatory treatment of atherosclerotic heart disease (94–97). MCC950, an NLRP3 inflammasome inhibitor, reduces infarct size and preserves cardiac function in a randomized, blinded translational large animal MI model (92). Thus, despite some unsolved mysteries, the results of the clinical research on colchicine, IL-1β antibodies, and IL-6Ra have undoubtedly led to a victory in the anti-inflammatory treatment of atherosclerotic heart disease. Under the influence of these three successes, the path of anti-inflammatory therapy for atherosclerotic diseases is flourishing.

**Table 2:** Major ongoing clinical trials involving anti-inflammatory agents in atherosclerotic heart disease.

**Trial name**	**Study design**	**Study population**	**Estimated enrollment**	**Estimated completion date**	**Intervention**	**Target**	**Primary outcomes**	**ClinicalTrials.gov identifier:**
**Therapy with a clear target**								
CLEVER-ACS trial	Phase I–II, randomized, paralleled, placebo-controlled trial	Patients with STEMI	150	December 31, 2020	Everolimus (7.5 mg for 3 days, followed By 5 mg for 2 days)	mTOR	Myocardial infarct size	NCT01529554
**Broad-spectrum anti-inflammatory approach**								
ALL-HEART study (93)	Multicenter, controlled, prospective, randomized, open-label blinded	Patients aged 60 years and older with ihd	5,215	Unknown	Allopurinol (up to 600 mg daily)	Multiple	Composite of nonfatal myocardial infarction, nonfatal stroke or cardiovascular death	ISRCTN32017426
COACS: Colchicine for Acute Coronary Syndromes	Phase III multicenter, double blind, randomized placebo-controlled	Patients with ACS (unstable angina or acute myocardial infarction)	500	Unknown	Colchicine 0.5 Mg/day vs. Placebo	Multiple	Overall mortality, new acute coronary syndrome, and ischemic stroke	NCT01906749
Colchicine and Spironolactone in patients with STEMI/SYNERGY Stent Registry	Phase III randomized placebo-controlled 4 study arms, 2 × 2 factorial design	Patients with MI/SYNERGY Stent Registry	7,000	March 30, 2025	Colchicine 1 mg/day And/or Spironolactone 25 mg/day and/or placebo and/or synergy stent	Multiple	Major Adverse Cardiac Events for SYNERGY Stent (defined as the composite of death, recurrent target vessel MI, stroke, or ischemia driven target vessel revascularization)	NCT03048825
CONVINCE	Phase III multicenter, Open-label, placebo controlled	Patients older than 40 years of age who have suffered an ischemic stroke or transient ischemic attack not caused by cardiac embolism or other defined causes	2,623	October 2021	Colchicine 0.5 mg/day vs. placebo	Multiple	Nonfatal recurrent ischemic stroke and coronary events and vascular death	NCT02898610

*CI, confidence interval; HR, hazard ratio; MI, myocardial infarction; STEMI, ST-segment elevation myocardial infarction; ACS, acute coronary syndrome; mTOR, mechanistic target of rapamycin; IHD, ischemic heart disease*.

## Author Contributions

All authors listed have made a substantial, direct and intellectual contribution to the work, and approved it for publication.

## Conflict of Interest

The authors declare that the research was conducted in the absence of any commercial or financial relationships that could be construed as a potential conflict of interest.
